# Juvenile Fibromyalgia and Headache Comorbidity in Children and Adolescents: A Literature Review

**DOI:** 10.1155/2019/3190829

**Published:** 2019-06-03

**Authors:** Emilia Matera, Roberto Palumbi, Antonia Peschechera, Maria Giuseppina Petruzzelli, Vittorio Sciruicchio, Marina de Tommaso, Lucia Margari

**Affiliations:** ^1^Child Neuropsychiatry Unit, Department of Basic Medical Sciences, Neurosciences and Sense Organs, University “A. Moro”, Piazza Giulio Cesare 11, 70100 Bari, Italy; ^2^Children Epilepsy and EEG Center, Via Aldo Moro 32, 70019 Bari, Italy; ^3^Neurology Unit, Department of Basic Medical Sciences, Neurosciences and Sense Organs, University “A. Moro”, Piazza Giulio Cesare 11, 70100 Bari, Italy

## Abstract

Juvenile fibromyalgia (JFM) is a chronic pain syndrome with onset in developmental age, characterized by widespread musculoskeletal pain associated with other neurological or nonneurological symptoms. Headache is one of the most frequent comorbid conditions with JFM, but this association is still poorly studied in the juvenile population. The literature review was conducted searching through PubMed, Scopus, and Web of Science with a combination of the following free-text terms: “fibromyalgia,” “juvenile fibromyalgia,” “headache,” “primary headache,” “migraine,” “children,” “adolescents,” and “comorbidity.” The research resulted only in two specific studies regarding comorbidity JFM + Juvenile Headache (JH). From each study, we extracted data about sample features, clinical characteristics of both JFM and PH, and assessment tools. The clinical approach to JFM and JH should include a complete examination of the main causes of comorbid diseases, thus improving the therapeutic approach to the patient in developmental age.

## 1. Introduction

Fibromyalgia is a condition characterized by chronic pain that prevails in adult population with a major prevalence between the ages 20–60 years and in females. The most frequent symptoms present in this syndrome are widespread and persistent pain, especially due to tender points, paresthesia, sleep disturbances, fatigue, irritable bowel, burning urination, headache, memory loss, difficulty concentration, and mood disorders [1].

In 1992, fibromyalgia was recognized by the World Health Organization as a debilitating disease, but its causes are not yet clear. If the difficulty in recognizing the causes, nature, and appropriate treatment of FM in adults is known, even less is known about the etiology and treatment of FM in developmental age. Few studies report rates of juvenile fibromyalgia (JFM) prevalence ranging from 2% to 6% of school-aged children [2–5]. As in the adult population, also in children and adolescents the disorder affects more frequently female than male subjects, and generally, JFM onset is reported during early adolescence, even if few cases are identified also in younger children [5, 6]. One of the main weaknesses of the literature data is that the diagnosis of JFM is based on the self-report symptoms of the American College of Rheumatology (ACR) diagnostic criteria of 1990 that are more appropriate for adults. Therefore, these studies could underestimate the true prevalence of JFM.

The diagnostic criteria proposed first by Yunus and Masi in 1985 and then by the ACR in 2010 introduce appropriate variations that seem to be more appropriate for the diagnosis of JFM [7].

In addition to the typical clinical symptoms of adult FM, in JFM are described some peculiar features, including joint laxity or hypermobility, a greater autonomic dysfunction, and specific psychopathological comorbidities, including depression and anxiety disorders [8–11]. Due to the chronic nature of the symptoms and the degree of pain and disability associated with FM in adulthood, early detection in children is of utmost importance; therefore, the aim of this review was to investigate on what has been studied on this topic so far in children and adolescents.

## 2. Methods

The literature review was conducted searching through PubMed, Scopus, and Web of Science with a combination of the following free-text terms: “fibromyalgia,” “juvenile fibromyalgia,” “headache,” “primary headache,” “migraine,” “children,” “adolescents,” and “comorbidity”; the search included studies published since the first description of the JFM till today.

The studies included in this research met the following criteria: (1) they should have a topic on JFM (clinical features and comorbidities) and headache; (2) patients enrolled should be children and adolescent; (3) they should be published in peer-reviewed journals. No other restrictions were applied.

## 3. Results

In the literature, only 4 studies met the research criteria. One study of 2014 was excluded because it involved adults with comorbid FM + Primary Headache (PH) and another study of 2013 was excluded because it made a comparison between two groups of young adults, affected, respectively, by PH and JFM. Only 2 studies ultimately analyzed the association between headache and JFM in patients under 18 years of age: one retrospective with a JFM sample and one observational with a chronic migraine sample. In Table 1 are reported the obtained results of the research, divided in years of study, type of study, number and age of participants, headache and JFM features, and assessment tools.

## 4. Discussion

Juvenile fibromyalgia (JFM) is commonly described as a noninflammatory chronic pain syndrome characterized by constant widespread pain, vegetative dysfunction, joint laxity or hypermobility, poor sleep quality, daytime sleepiness, and an altered mood. At present, in contrast with the adult population, in which FM and headache—mostly chronic primary headache—are often comorbid conditions [15–17], there are few studies about the comorbidity between fibromyalgia and migraine in the pediatric population.

Some studies report that FM clinical outcome is negatively influenced by the comorbidity with headache and that both disorders are characterized by a worst impact on the quality of life.

In 2007, Eraso et al. studied retrospectively a sample of 148 patients affected by JFM; 118 out of 148 subjects presented headache in comorbidity. They divided these patients in two groups on the basis of the JFM onset (under or above 10 years of age); then, comparing the prevalence of the headache comorbidity between the two groups, no statistically significant difference was found (*p*=0.8) [12]. In 2017, de Tommaso et al. studied 151 patients affected by PH (47 chronic headache; 92 migraine without aura; 12 migraine with aura). Only 5 out 151 patients (0.03%) fulfilled the criteria for JFM [13]. These results suggest that even if headache could be not only one of the most frequent onset symptoms of JFM, chronic primary headache is not a frequently associated condition, unlike what happens in adult population.

Moreover, when it comes to the comorbidity between headache and JFM, the symptoms of central sensitization or sleep disturbances were other topics frequently discussed.

Eraso et al. showed that JFM children had lower thresholds for tenderness and that the number of painful tender points was strongly correlated with distress, including sleep disturbance [12]. de Tommaso et al. underlined that PH + JFM children presented more evident allodynia and pericranial tenderness and symptoms of central sensitization compared to subjects with PH only; sleep disturbances did not differ significantly between children with PH + JFM and JH only, but the short sleep duration was a facilitator of the central sensitization. Therefore, the comorbidity between JFM and PH might be related to common neurobiological basis, including the central mechanisms of pain regulation [13].

In adult population, several authors include FM and headache (tension type and migraine) in the central sensitivity syndromes and chronic pain disorders such us whiplash, irritable bowel syndrome, temporomandibular syndrome, low back pain, restless legs syndrome, myofacial pain syndrome, chronic fatigue syndrome, and osteoarthritis [14, 18–22]. Central pain sensitization is a process characterized by a generalized hypersensitivity of the somatosensory system, and it reflects not only an increase in the ascending pathways of pain but also an alteration of the descending pain inhibitory pathways, resulting in a dysfunction of the endogenous analgesic control. Moreover, several studies describe an increase of the activity in brain areas that are involved in the perception of acute pain (insular cortex, anterior cingulate cortex, and prefrontal cortex) and in regions generally not involved in the sense perception of acute pain (lateral dorsal frontal cortex and parietal associative cortex) [23].

Soee et al. showed that children with chronic tension-type headaches have an altered perception of pain after the application of increasing intensity pressure on the trapezius and temporal muscles. They hypothesized that this reaction could be probably related to the central sensitization [24].

The central sensitization is interpreted as a consequence of the muscle-cutaneous damage in FM or of the activation of the trigeminal vascular system in headache, which induces an inflammation followed by peripheral and central sensitization, responsible for the persistence of pain [13, 25–27].

Studies investigating the characteristics of adult subjects with FM and headache in comorbidity [13, 28, 29] reported higher frequency and intensity of headache, anxiety levels, depressive symptoms, pericranial tenderness, sleep disturbances, and reduced physical performances compared to patients without comorbidity. In children, Eraso et al. [12] reported frequent symptoms of generalized pain, fatigue, sleep disturbances, and stiffness, while de Tommaso et al. [13] showed relevant depressive symptoms, pericranial tenderness, and allodynia. If the differences compared to adults seem to be related to the psychopathological traits (anxiety symptoms more relevant in adults and depressive symptoms more relevant in children), the differences between the results of the two studies carried out in children could be related to the different methods of recruitment of the samples and the aims of the studies.

Although it seems that prognosis of JFM is better in children than in adults [3], it should be considered that the comorbidity with headache negatively impacts the domestic, scholastic, and social functioning of the affected subjects, creating a vicious circle that on the one hand can exacerbate the FM/headache syndrome and on the other it can create the basis for the persistence of psychopathological disorders, even many years after the diagnosis [5, 10, 30].

Further prospective studies on children and adolescents affected by JFM and PH are necessary to better clarify the current limited evidence, especially to make the treatment more specific in the case of comorbidity, considering that, in young patients, the pharmacological therapeutic options seem fewer than the ones available for the adult population.

## 5. Conclusion

In the near future, the clinical approach to JFM should include a complete examination of the main causes of comorbid diseases, and in patients suffering from JH, it should evaluate the accompanying symptoms (anxiety, sleep disorders, and widespread pain) that may be risk signs of a comorbidity with FM, thus improving the therapeutic approach to the patient in developmental age considering the chronic nature of the symptoms and the degree of suffering and disability associated with this condition in adulthood.

## Figures and Tables

**Table 1:** Features of the studies reviewed.

Study	Type of study	Number of participants	Sample features	JFM features	Headache features	Assessment tools
Eraso et al. [12]	Retrospective	Total: 148	JFM diagnosis Two group (A and B) on the basis of the age of the onset (< or >10 years of age) For each group, the following were analyzed: mean age at the onset, mean interval between mean age at the onset and mean age of the diagnosis, clinical features	Onset (age and symptoms) Tender points Laboratory test (complete blood count, antinuclear antibody test) Management and outcome	Not investigated	FMS assessment: specifically designed format to collect pertinent information Joint hypermobility: Carter and Wilkinson criteria
		118 out of 148 were affected by PH and JFM				

de Tommaso et al. [13]	Observational cross-sectional	Total: 151	Primary headache patients Patients were divided into 4 categories of frequency (1–4; 5–9; 10–14; 15–30 days/month)		Migraine onset (months) Frequency of headache (days/migraine/month) Type of primary headache: chronic migraine, migraine without aura, migraine with aura Allodynia Pericranial tenderness Pain catastrophizing	Allodynia: symptoms checklist reported by Ashina et al. [14] JFM symptoms: widespread index, symptom severity index; quality of life: migraine disability (Ped MIDAS); PedsQL; parent proxy report Sleep assessment: sleep disturbance scales for children Pain catastrophizing: pain catastrophizing scale for children
		5 out 151 were affected by PH and JFM				
